# Exploring College Students’ Acceptance of and Behavioral Intentions Toward Different Sorghum-Based Foods

**DOI:** 10.3390/foods14061065

**Published:** 2025-03-20

**Authors:** Oak-Hee Park, Andrea Sosa-Holwerda, Surya Raj Niraula, Krithika Maki, Leslie Thompson, Naima Moustaid-Moussa

**Affiliations:** 1 Department of Interdisciplinary Human Sciences, College of Health and Human Sciences, Texas Tech University, Lubbock, TX 79409, USA; 2 Department of Nutritional Sciences, College of Health and Human Sciences, Texas Tech University, Lubbock, TX 79409, USA; andrea.sosa@ttu.edu (A.S.-H.); surya.niraula@ttu.edu (S.R.N.); naima.moustaid-moussa@ttu.edu (N.M.-M.); 3 Department of Animal and Food Sciences, Davis College of Agricultural Sciences & Natural Resources, Texas Tech University, Lubbock, TX 79415, USA; kmaki@ttu.edu (K.M.); leslie.thompson@ttu.edu (L.T.); 4 Obesity Research Institute, Texas Tech University, Lubbock, TX 79409, USA; 5 Institute for One Health Innovation, Texas Tech University Health Sciences Center, Texas Tech University, Lubbock, TX 79409, USA

**Keywords:** sorghum, whole grain intake, sensory evaluation, nine-point hedonic scale, college students, acceptance, eating and purchase intentions

## Abstract

Sorghum is a nutritious, healthy, gluten-free whole grain, with the United States (U.S.) leading its production globally. While sorghum is consumed worldwide, it is mainly used for animal feed and biofuel in the U.S. Organoleptic characteristics and consumers’ perceptions determine food acceptance and eating behavior. Therefore, this study aimed to investigate the acceptance of and eating and purchase intentions toward sorghum-based foods among college students in a southern university in the U.S. Eighty-three students participated in a series of sensory evaluations using two sets of four sorghum samples each and a 15 min break. Seven sensory attributes were evaluated with a nine-point hedonic scale, and a five-point scale was used for eating and purchase intentions. To assess the panelists’ acceptance, the overall acceptance scale score (range: 1–9) was normalized (range: 0–100) and used for analyses. Spiced sorghum cookies (77.95 ± 14.23) had the highest acceptance, followed by sorghum shrimp grits (74.51 ± 19.42). Overall acceptance, eating intention, and purchase intention were strongly associated across all food items, although the strength differed by food type. Sorghum-based foods were accepted despite the participants’ lack of exposure to sorghum and its null consumption. These outcomes will help to develop innovative sorghum-based foods to facilitate sorghum consumption and benefit consumer health in the U.S.

## 1. Introduction

### 1.1. College Students’ Whole Grain Eating Habits

As college transition begins, students start to adjust to new eating habits. Regardless of their understanding of nutrition and health, their ability to choose various foods influences their eating practices [1]. College students often face challenges when making food decisions because of many circumstances: leaving their homes, adjusting to a new life, financial and time constraints, convenience, and food preferences [1]. Beyond these variables that impact students’ food choices, the food environment on campus is limited. While healthy options are promoted, they are often expensive and less available or accessible compared to unhealthy options, such as ultra-processed foods and/or fast foods [2,3,4].

According to the Dietary Guidelines for Americans (DGA) 2020–2025 [5], at least half of the grains (or 3 servings/day) consumed daily by an adult should be whole grains. However, whole grain consumption in a given day accounts for only 15.8% of an adult’s total grain consumption [6,7]. For example, on average, less than one serving of whole grains per day is usually consumed by American college students [8], with cereals and breads as their main whole grain sources, indicating that they are not meeting the DGA recommendations [6]. When unhealthy eating behaviors are adopted, health problems may develop [9] as a consequence of worsening dietary habits [1], which may increase health risks later in life [8].

### 1.2. Sorghum: Nutrients, Health Benefits, Production, and Human Consumption

Sorghum is a gluten-free whole grain rich in nutrients, dietary fiber, and phytochemicals. Its nutritional content is based on carbohydrates, protein, and lipids (65–80 g, 7–15 g, 1.5–6 g, respectively, in every 100 g of sorghum) depending on the variety. It is also a good source of complex B vitamins, fat-soluble vitamins, and polyunsaturated fatty acids (i.e., linoleic, oleic, palmitic, and linolenic acids) [10]. Flavonoids and condensed tannins are its main phenolic compounds, which are the phytochemicals’ biggest group [11]. Phytochemicals are naturally found in plants. Disease prevention related to fruits and vegetables is associated with plants’ phytochemicals, since they act as antioxidants and reduce inflammation, cell mutation, and the occurrence of several diseases [12]. Among sorghum’s phytochemicals, anthocyanins are related to reducing the risk of cardiovascular diseases, lowering lipid levels in the blood, and providing anti-diabetic benefits [12].

Sorghum’s nutritional characteristics are comparable to those of wheat, and its chemical composition can be compared to that of maize, wheat, and rice [13]. However, its distinctive bioactive features and phenolic composition are rarely found in other grains. When compared to wheat and millet, sorghum shows more bioactivity against cancer [12], because the phenolic composition of sorghum may reduce oxidative stress, which results from an imbalance between free radical production and antioxidant defense [11]. In addition to its nutritional profile, it is a gluten-free grain, making its consumption suitable for individuals with celiac disease.

Sorghum is an ancient grain native to northeastern Africa and was first introduced into the U.S. by Benjamin Franklin in the late 1700s for use in broom making. By the early 1900s, sorghum was used in different ways: plants used as forage, grains cooked whole or ground into flour, stalks crushed to make syrup, and tassel used for making brooms and wallboard [14].

Today, sorghum is the fifth leading crop worldwide, and the U.S. is the main sorghum producer in the world [15]. Sorghum’s exceptional nutritional and agronomic characteristics make it drought-resistant with high adaptability to harsh climates, allowing it to grow in arid and semi-arid conditions, demanding little water to grow [16]. Moreover, it is highly sustainable due to its low production cost and higher net returns [17,18]. The gluten-free and non-GMO (genetically modified organism) characteristics of sorghum grain have received attention from the food industry, highlighting the potential of sorghum as a major ingredient in new foods [12].

However, in Western countries, like the U.S. and Canada, sorghum has been mainly used in animal feed and biofuel production, and only 3% of the sorghum produced is used for human consumption in the U.S. [16,19]. For instance, compared to oats, rice, and other grains, sorghum has been used for relatively few products such as pasta, bread, syrup, and popped sorghum, or as a minor ingredient in ready-to-eat breakfast cereals/bars [12,20,21].

In contrast, about forty percent of the sorghum produced is utilized for human consumption in India, Africa, China, and other low-income regions [17]. Sorghum foods in these countries/regions include types of bakery products (e.g., roti, dosa, and injera), porridges (e.g., sankati, ogi, and ugali), beverages (e.g., pito and baijiu), breakfast (e.g., upma), snacks (e.g., popped sorghum), and part of main meals (e.g., annam, nufro, and okbaba) [12,22].

### 1.3. Reasons for Conducting Sensory Evaluation of Sorghum-Based Foods

Despite its favorable nutritional and health benefits and high production in the U.S., sorghum is still considered to be of low value for human consumption as part of a regular diet [23]. The limited availability of sorghum products in the food retail market might lead to poor consumer recognition of sorghum as a human food, so consumers do not know what sorghum is and how to incorporate sorghum into their regular meals. This emphasizes the importance of developing sorghum-based foods to facilitate sorghum consumption [21]. Further, there is limited research on the acceptance of sorghum-based foods (that is not a single product) using sensory evaluation among U.S. consumers [24].

Previous studies on consumers’ acceptability of sorghum-based foods through sensory analysis were heavily focused on single products: cookies developed from 12 sorghum cultivars [25] and from sorghum and cowpea flours [26], biscuits made from sorghum and bread wheat–soy composite [27], gluten-free sorghum breads using flours of selected sorghum genotypes [28], extruded sorghum breakfast cereals [29], and pasta/spaghetti [30] with different formulations of grains/grains with other food materials (i.e., soy protein) to meet organoleptic quality.

Incorporating sorghum into meals that are commonly consumed and easy to add to the diet is a strategy used not only to promote sorghum consumption, but also to reach a large population [17]. When new food products are launched to the market, conducting a sensory evaluation beforehand is essential to determine its acceptance (or rejection) among the target population [31]. Organoleptic characteristics are vital in food products since they will influence consumer expectations, acceptability, and purchase and eating intentions; therefore, it is crucial to understand the significance of the sensory analysis of sorghum-based foods [23]. Sensory analysis is especially critical when the food product that is being tested is not part of the current diet [28], which is the case of sorghum in the U.S.

To the best of our knowledge, there are no studies investigating the acceptance of various sorghum-based foods considered as regular meals and eating and purchase intentions through consumer sensory analysis in the U.S. Therefore, the objectives of this study are 1) to explore the sensory qualities of sorghum-based foods to confirm consumer acceptance and 2) to explain the relationships among acceptance, eating, and purchase intentions for sorghum-based foods. The success of this study will open the door to create new sorghum-based foods that can be broadly accepted and applied as human food; build a new culture of eating sorghum as regular meals; and provide a sustainable solution to reduce the risks of chronic diseases and food insecurity.

## 2. Materials and Methods

### 2.1. Data Collection

The sensory evaluation was conducted in collaboration with Texas Tech University’s Hospitality Services at Wiggins Complex venue, where the data were collected. This study was approved by the Institutional Review Board (IRB) of Texas Tech University (IRB2022-701).

### 2.2. Raw Sorghum Characterization

Sorghum grains used in all recipes were obtained from Richardson Seeds Ltd. (Vega, TX, USA) and a farmer collaborator. Other food materials were purchased from a local supermarket (Lubbock, TX, USA). White sorghum was found to be the most suitable for cooking conditions and sorghum food development. Either ground sorghum or entire sorghum was used for this study (Table 1).

### 2.3. Menu Development and Selection

Twenty-five sorghum recipes were developed by an Executive Chef and a Sous Chef of Texas Tech University’s Hospitality Services. The menu included eight entrées, four soups, two sides, three salads, two snacks, four baked items, and two breakfast recipes. Nine food experts, including a registered dietitian, three food and nutrition scientists, three college students trained for sensory evaluation, and two adults who work for the sorghum food industry, evaluated the twenty-five sorghum-based foods and then selected final eight items based on affordability, sensory attributes, and replicability. The eight selected foods and sorghum formats used for each individual recipe are shown in Table 1.

### 2.4. Menu Preparation

All recipes were prepared differently according to the food type (i.e., main dish, salad, dessert, and breakfast) and sorghum format (i.e., ground sorghum, intact popped grain, intact grain, and flour). Table 1 describes the preparation of each menu item in detail.

### 2.5. Recruitment

Texas Tech University’s email announcement was used to recruit participants. Undergraduate students who were interested in this study were invited to fill out a Qualtrics (Qualtrics, Provo, UT, USA) online questionnaire (embedded in the email announcement) to determine their eligibility. The inclusion criteria were based on an age ≥ 18 years; grain consumers with no dietary restrictions (e.g., vegan or vegetarian), no food allergies, and/or sensitivities to milk, peanuts, egg, soy, wheat, and tree nuts; and they had to be enrolled at Texas Tech University. Because olfactory and taste senses are compromised when infected with COVID-19 or a common cold, the participants were asked to reschedule two weeks after experiencing symptoms to avoid the inability to accurately evaluate the food samples. Students who did not meet these criteria were excluded from the study. Study information and a consent form were sent to each participant via email, and signed consent forms were collected before the sensory evaluation. A total of eighty-three students participated in the study, which provided 85% power to detect 9% of difference in the scores between the groups with a maximum variability of 19.42%.

### 2.6. Food Sample Preparation

A three-digit individual code number label was printed to code the food containers after the sorghum samples had been randomly assigned to a number. Containers of neutral color and made of inert material were used to avoid off flavors and odors not related to the food sample. The temperature of each sorghum sample was adjusted according to the type of food item that was served. Samples were served at a standardized amount of 2 oz [32].

### 2.7. Sorghum Sensory Evaluation and Behavioral Intention Measures

The sorghum sensory evaluation was conducted at Texas Tech University’s Hospitality Services at Wiggins Complex venue with a capacity for 30 people and a controlled temperature of 75 ± 2 °F. Each participant had a test divider to avoid bias among panelists. Prior to the evaluation, instructions were given to the panelists regarding palate cleansing, the use of the scoring sheets, and a short explanation about organoleptic characteristics; the definition and explanation of whole grains was also given to the participants for each of the four sessions that were held. The participants were also asked not to eat, drink coffee, or smoke an hour before the sensory analysis and not to discuss food samples with other participants during the session and break.

Each session was conducted within 24 h of the sorghum menu preparation. The eight sorghum samples were served to each participant following a randomized order. The sorghum samples were served monadically; deionized water and unsalted crackers were provided to the panelists for palate cleansing before tasting each sample to avoid a residual taste from the previous sample. The panelists rated seven organoleptic characteristics (i.e., appearance, aroma, texture/mouthfeel, taste, flavor, sweetness, and overall acceptance) using a 9-point hedonic scale ranging from 1 = dislike extremely, 2 = dislike very much, 3 = dislike moderately, 4 = dislike slightly, 5 = neither like nor dislike, 6 = like slightly, 7 = like moderately, 8 = like very much to 9 = like extremely. To avoid sensory and mental fatigue, after tasting the first set of four sorghum samples with 5 min in between each sample, the panelists had a 15 min break before rating the second set of four sorghum samples. The sensory evaluation process and measurements were followed by recommendations from published literature and studies [32,33,34,35,36].

In addition to the acceptance of each menu item, eating and purchase intentions were determined using a 5-point Likert scale (1 = definitely would not eat, 2 = probably would not eat, 3 = neutral, 4 = probably would eat, 5 = definitely would eat), as described by published studies [37,38]. Data were collected using scoring sheets for both sensory and eating and purchase intention evaluations.

### 2.8. Demographic Questionnaire and Food Frequency

All questions for the demographic questionnaire and validated food frequency questionnaires (FFQs) were adopted from previously published studies [6,39,40,41]. The demographic questionnaire covered gender, age, college year, ethnicity, and frequency of using the university’s dining services [6]. To understand college students’ whole grain consumption and their choices, a validated FFQ [39] and a whole grain choice question (i.e., which of the following whole grain/whole grain products do you consume at least one a week?) with 13 whole grain types were given to the participants [20,41]. A FFQ consists of a food list that is intended to indicate the usual intake of food over a period of time [39]. The FFQ instrument used for this study was a validated Harvard University FFQ, specifically, the whole grain food listed in the section of breads, cereals, and starches [40]. The FFQ required the participant to specify their typical frequency of intake for a given period of time (i.e., never or less than once per month, 1–3 per month, 1 per week, 2–4 per week, 5–6 per week, 1 per day, 2–3 per day, 4–5 per day, or more than 6 per day) for each of the foods listed, with the aim of obtaining an estimate of the average daily total intake [42].

### 2.9. Content Analysis

The participants were asked to answer an open-ended question to describe their perceptions of each sorghum sample in addition to the specific organoleptic characteristics that were assessed using the 9-point hedonic scale. Based on the published recommendations of qualitative method analysis [43,44], the collected qualitative data were reviewed individually by three of the authors, and then initial codes were obtained to create a list of codes for each sorghum sample. Consensus discussions of each code were carried out to represent the overall descriptions for each sample. Frequency and visualization of the qualitative data were completed using NVivo (14.0) [45].

### 2.10. Statistical Analyses

The collected quantitative data were reviewed by three authors of this study and underwent data cleaning, as recommended by published studies [46,47]. Missing data were captured and coded as “999” in the data set, and duplicated responses were checked and corrected. If a participant skipped more than 10% of the total questions, then their data were removed from the data set. All three authors conducted a final review of the data set to ensure the high quality of the data before statistical analyses.

The data were analyzed with IBM SPSS version 29.0.0.0 (241). Descriptive statistics were used to describe demographic characteristics as well as the sensory attributes and eating and purchase intentions of the participants in terms of mean, standard deviation, median, and percentage. Cross-tabulation was conducted to examine the association between ordinal categorical acceptance and the different sorghum-based menu items. A Shapiro–Wilk test showed significant results during the checking of normality in terms of the acceptance score for different meal groups thus, it was converted into normal distribution after inverse transformation. One-way analysis of variance was considered for more than two categories, and *t*-test was used for two categories. The General Linear Model with Bonferroni correction was applied to examine the fixed effect, adjusting the effects of possible confounders. Spearman correlation was performed to determine the associations between the overall acceptance of sorghum-based foods and eating and purchase intentions. The probability of significance was set at 5%.

## 3. Results

### 3.1. Descriptive Results

Eighty-three participants evaluated the eight different sorghum-based menu items. Almost 58% of the participants were female. The participants were aged between 18 and 35 years old. Around one-third of the participants were Non-Hispanic White (31.3%), followed by Hispanics or Latinos (26.5%) and Asians (25.3%). Over 78% of the participants used the university dining services, while 15.7% of the participants never used the services (Table 2).

### 3.2. Whole Grain Consumption

**Figure 1:**
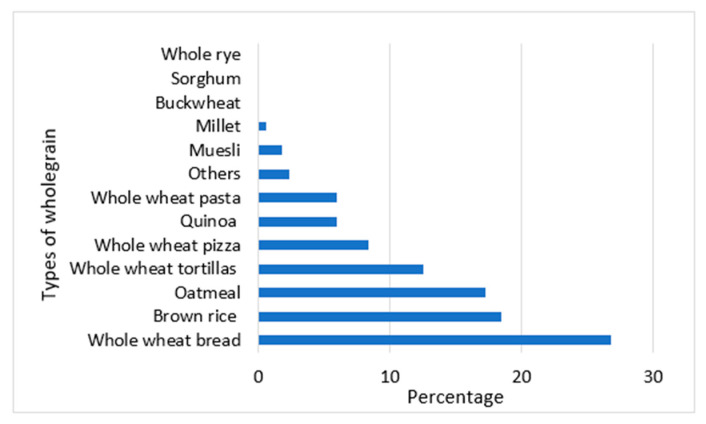
Proportion of whole grain types consumed weekly by participants.

Whole wheat bread, brown rice, and oatmeal were the most frequently consumed whole grains for weekly consumption (Figure 1). The average daily servings (in the past 7 days) of whole grain was 0.69 ± 1.02. The weekly frequency of whole grains consumed according to gender was higher for males (2.25 ± 1.38) than females (1.81 ± 1.44). Sophomore students had the highest weekly frequency (2.38 ± 1.54), while seniors had the lowest (1.74 ± 1.36). Asians exhibited the highest frequency per week (2.24 ± 1.7), while White and Non-Hispanic or Latino (1.88 ± 1.36) showed the lowest frequency.

### 3.3. Content Analysis

**Figure 2:**
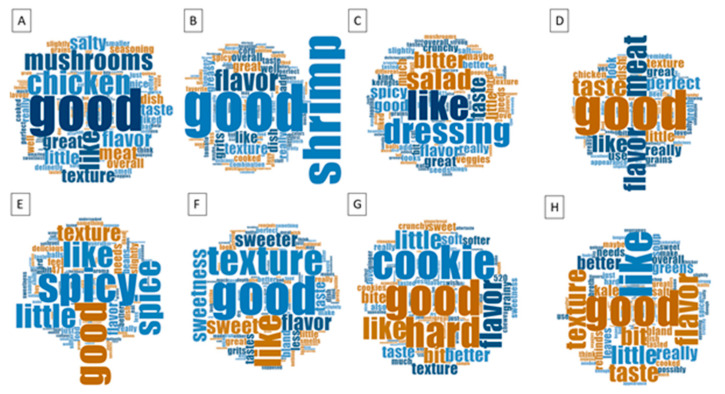
Word clouds for each sorghum-based menu item. (**A**) Chicken with sorghum, (**B**) sorghum shrimp grits, (**C**) arugula salad with popped sorghum, (**D**) beef sorghum, (**E**) sorghum vegan chili, (**F**) sweet sorghum grits, (**G**) spiced sorghum cookie, and (**H**) kale sorghum soup.

The frequency of the words determined their sizes in each word cloud shown in Figure 2. The higher the frequency of a word, the bigger the word is shown in the overall description of each sorghum-based food. The most common word used for all sorghum-based menu items was “good”, with the exception of the arugula salad with popped sorghum. Descriptors such as “like”, “flavor”, and “texture” were used for the kale sorghum soup, spiced sorghum cookie, sweet sorghum grits, beef sorghum, sorghum shrimp grits, and chicken with sorghum. “Spicy” was used only for the sorghum vegan chili, and “hard” was a notable descriptor for the spiced sorghum cookie. Other ingredients that were used in the recipes were also noted. For instance, “mushrooms”, “shrimp”, and “dressing” were ingredients that were used in menu items A, B, and C, as described in Figure 2.

### 3.4. Sensory Evaluation

Normal conversion of the sensory evaluation (i.e., overall acceptance attribute) data was applied for statistical analysis purposes (see Table 3, Table 4 and Table 5). Hedonic scales allow participants’ responses to be transformed into numbers, resulting in a score from 0 to 100 [48]. The mean acceptance score of all sorghum-based menu items was over 60 (Table 3). The arugula salad with popped sorghum (60.37 ± 16.77) had the lowest acceptance. In contrast, the most accepted item was the spiced sorghum cookie (77.95 ± 14.23), followed by sorghum shrimp grits (74.51 ± 19.42), chicken with sorghum (73.61 ± 14.46), and beef sorghum (73.36 ± 14.44).

**Figure 3:**
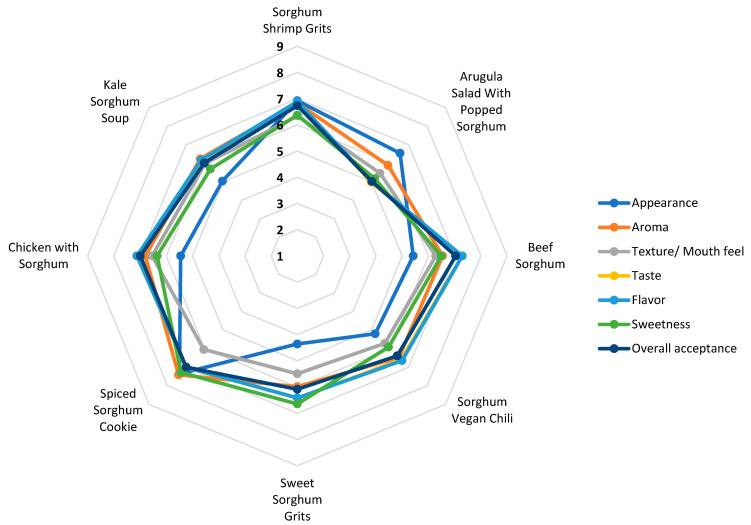
Sensory evaluation description of sorghum-based foods using a 9-point hedonic scale ranging from 1 = dislike extremely, 2 = dislike very much, 3 = dislike moderately, 4 = dislike slightly, 5 = neither like nor dislike, 6 = like slightly, 7 = like moderately, 8 = like very much to 9 = like extremely.

Figure 3 displays the evaluation of seven sensory attributes of the eight sorghum-based foods using a nine-point hedonic scale. The further the point is from the center, the higher the value for that attribute, whereas the values that are closer to the center represent a lower score. The spiced sorghum cookie and sorghum shrimp grits illustrate this clearly. The spiced sorghum cookie has the highest score (between 7 = like moderately and 8 = like very much) for almost all attributes, except for texture/mouthfeel, with a score close to 5, meaning neither like nor dislike. Similarly, sorghum shrimp grits had higher scores across all attributes, indicating like moderately. Among the seven attributes, the appearance attribute received the lowest score, especially sweet sorghum grits (close to 4 = dislike slightly).

The bivariate analysis of the acceptance score was not significantly associated with the age (t = 0.299, *p* = 0.765) or ethnic group of the students (F = 1.882, *p* = 0.131). However, the score was significantly associated with college year (F = 6.736, *p* < 0.001) and gender (t = −2.948, *p* = 0.003). These significant effects were adjusted in the General Linear Model to identify the real association of different meals on the acceptance score (Table 4). All sorghum-based menu items were included, using kale sorghum soup as the reference category. For gender and college year, female and seniors were the reference categories, respectively. Chicken with sorghum (β = 8.725, *p* < 0.001), sorghum shrimp grits (β = 9.622, *p* < 0.001), beef sorghum (β = 8.479, *p* < 0.001), and spiced sorghum cookie (β = 13.015, *p* < 0.001) exhibited a significant increase in the acceptance score. In contrast, the arugula salad with popped sorghum exhibited a significant decrease (β = −4.881, *p* = 0.042) in the acceptance score. Sorghum vegan chili and sweet sorghum grits had no statistically significant effect.

**Figure 4:**
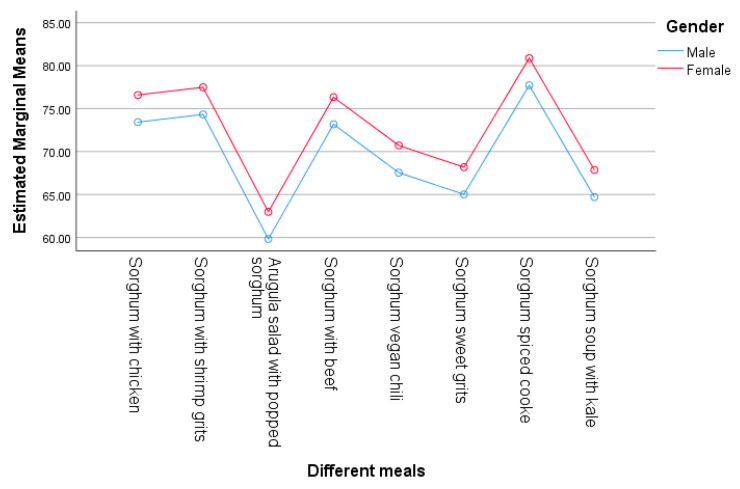
Estimated marginal means for acceptance score by gender.

**Figure 5:**
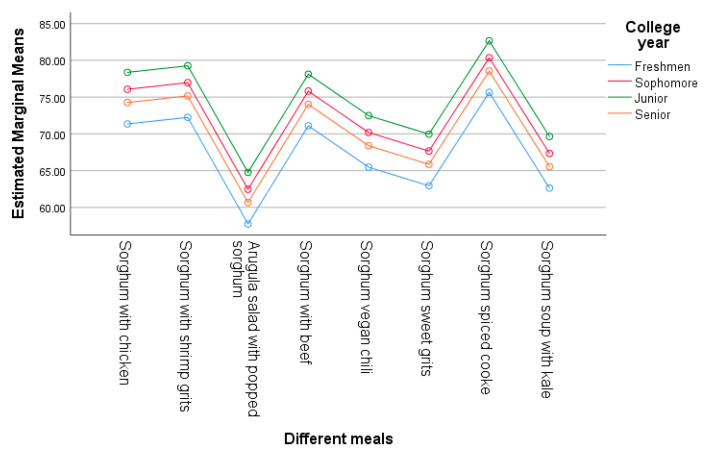
Estimated marginal means for acceptance score by college year.

When compared to females, males had a significant lower (β = −3.162, *p* = 0.011) acceptance score. Junior students exhibited a significant increase (β = 4.101, *p* = 0.03) compared to the other college years. Freshman students had lower scores; however, this was not significant (β = −2.917, *p* = 0.065). These results suggest that both gender and college year have an effect and contribute significantly to the variability in the acceptance of sorghum-based foods, as shown in Table 4 and Figure 4 and Figure 5. The estimated marginal means (Figure 4 and Figure 5) provide the average score of each sorghum-based menu item, showing the participant acceptance with the lowest mean of 61.40 for arugula salad with popped sorghum and the highest for the spiced sorghum cookie (79.29).

Table 5 displays the pairwise association of acceptance scores over the sorghum-based foods after Bonferroni correction. There were significant differences between several sorghum-based food pairs. For example, sorghum shrimp grits and arugula salad with popped sorghum were significantly different (*p* < 0.001), but sorghum shrimp grits and chicken with sorghum were not significantly different. Pairs of sorghum-based foods with significant differences included A-C, A-F, A-H, B-C, B-F, B-H, C-D, C-E, C-G, D-F, D-H, E-G, F-G, and G-H. On the other hand, several pairs of sorghum-based foods such as A-B, A-D, A-E, A-G, B-D, B-E, B-G, C-F, C-H, D-E, D-G, E-F, E-H, and F-H were not significantly different. In particular, arugula salad with popped sorghum exhibited more significant differences compared to the other sorghum foods, except sweet sorghum grits (*p* = 0.854).

### 3.5. Eating and Purchase Intentions

Spearman’s rank correlation was run to assess the relationships between overall acceptance and eating and purchase intentions for all eight sorghum-based menu items. The item with very strong, positive correlations across all variables was sorghum shrimp grits (0.864 ≤ *r*_s_ ≤ 0.886, *p* < 0.01). Beef sorghum (0.706 ≤ *r*_s_ ≤ 0.773, *p* < 0.01), sorghum vegan chili (0.758 ≤ *r*_s_ ≤ 0.791, *p* < 0.01), spiced sorghum cookie (0.649 ≤ *r*_s_ ≤ 0.724, *p* < 0.01), chicken with sorghum (0.633 ≤ *r*_s_ ≤ 0.772, *p* < 0.01), and kale sorghum soup (0.665 ≤ *r*_s_ ≤ 0.749, *p* < 0.01) exhibited strong, positive correlations across all variables (Table 6).

## 4. Discussion

### 4.1. Whole Grain Consumption: Limited and Not Meeting DGA Recommendations

The most frequently consumed whole grain products by the participants were limited to mainly three products: whole wheat bread, brown rice, and oatmeal, indicating a low variety of whole grain consumption. Also, the participants in this study consumed less than one serving of whole grain (0.69 ± 1.02) per day. This is of importance because individuals who do not consume enough whole grains are likely to be deficient in some essential nutrients and the positive health effects of whole grains are determined by the frequency of their consumption [41]. These results are similar to those reported by Hicks-Roof et al. [6], where 70% of college students stated that the two main whole grain products they consumed were limited only to breakfast cereal and bread. These eating patterns may be explained by Western countries’ diets, which are high in processed foods and have excess sugar and fat, and are associated with a reduced nutrient quality [49,50]. The U.S. is not an exception; most of the grain products consumed are highly processed with a low content of micronutrients and minimal to no whole grain content [51,52].

The Dietary Guidelines for Americans (DGA) have recommended whole grain consumption (≥50% daily intake) since 2005 [53]. Nevertheless, by 2016, whole grains made up only about 15.8% of an adult’s daily grain consumption [6,7], and our result also revealed a similar outcome, confirming that the DGA’s whole grain intake recommendation is still not being met. In our study, we did not ask the reasons for not choosing whole grain foods. However, other studies [54,55] found that a lack of whole grain knowledge, poor sensory qualities, high prices, and poor availability outside the home were barriers to whole grain consumption among college students. Over 65% of the participants in our study used the university dining services on a daily and weekly basis. This finding indicates the need for whole grain foods to be made available on the campus. In other words, if more whole grain foods are available in dining services, it will promote students’ experiences such as tasting that might result in the regular consumption of whole grain foods like sorghum-based foods.

### 4.2. Content Analysis: Good Sensory Acceptance with Lower Texture Descriptors

For a product to be selected, eaten, and purchased, the sensory characteristics are crucial [56]. In addition to sensory evaluation using a hedonic scale, this study explored opinions and perceptions on various sorghum-based foods using word clouds. The most common word that was found to describe all eight sorghum-based menu items was “good”, showing the food’s acceptability. “Flavor”, “taste”, and “like” were other descriptors that were common across the sorghum samples, indicating their acceptability. Nonetheless, “spicy”, “texture”, and “hard” were other words expressed by the participants, indicating opportunities to improve the sorghum-based recipes. As expected, “texture” appeared in all samples. This correlates with our results shown in Figure 3, where texture/mouthfeel is located towards the center of the radar graph, representing a lower score among the attributes.

Gluten is a protein that confers viscoelasticity and water-binding properties, which are desirable characteristics in food products. Because sorghum is gluten-free, it does not have the aforementioned characteristics [57,58]. Moreover, the hardness of a whole grain is due to the lack of a milling process and the fact that is has three layers, whereas a refined grain only contains the endosperm or the starchy part of the grain, providing a smoother texture and more acceptable sensory attributes [56,59]. Gondal et al. [56] reported similar results when participants noted the hardness of whole grains (i.e., brown rice) compared to refined grains (i.e., white rice varieties). This highlights the important role that texture plays, because food acceptance also depends on texture/mouthfeel attributes [60]. Sorghum’s granularity and harder texture can be improved by utilizing food processing techniques that allow sorghum whole grains to resemble the texture of refined grain to increase its acceptance [61]. These descriptors provide the starting point for the future development of sorghum-based foods by addressing the appropriate sensory characteristics that the consumer wants.

### 4.3. Sensory Evaluation: Guidance for Sorghum Applications

The goal of sensory analysis is to measure the sensory qualities/attributes (e.g., texture, aroma, appearance) of food by humans to determine consumer acceptance of the food. In this field, humans are the measuring device, and food is what is to be measured, making sensory analysis unique and clearly different from the chemical and machine analysis of food items [62]. Likewise, one of the main goals of developing a highly nutritional food is creating it with generally accepted sensory attributes, especially when it comes to whole grain products with a high fiber content [17].

Although the sensory attributes of some sorghum products such as biscuits and cereals have been studied, there is limited research that has tested sorghum as a whole food [6]. This study focused on the acceptance of the previously mentioned sorghum-based recipes through sensory analysis. All of the recipes had a total acceptance score of over 60%, indicating acceptability, with half of the items scoring over 70%. This is of vital importance, because gluten-free products tend to have lower sensorial characteristics compared to foods containing gluten [63].

According to Pechey et al.’s study [64], vegetarian foods tend to be less accepted and selected than foods containing meat. This aligns with our findings. In detail, the less preferred sorghum-based menu items did not have any type of meat, whereas three of the four most preferred items had different types of meats such as chicken, beef, and shrimp. Despite the spiced sorghum cookie’s hardness described by the participants, it obtained the highest acceptance score overall and was perceived to be sweeter than the sweet grits. Moreover, the majority of herbivores and omnivores instinctively prefer sweet meals [65]. Agreeing with this, it was also found that young adults with higher education consumed more sweet foods like cookies and cakes [66]. This may also explain why the spiced sorghum cookie had the highest acceptance among our target population.

Figure 3 visually reflects that our results from the acceptance scores align with the overall acceptance using the nine-point hedonic scale. For instance, we can clearly see that the arugula salad with popped sorghum had the lowest overall acceptance, as presented in Table 2. The lower acceptance of arugula salad might be attributed to arugula’s bitterness itself. However, a previous study by Aguiar et al. [28] explained that sorghum might have an aftertaste that may be perceived as bitter due to sorghum’s high content of tannins, which are highly associated with bitterness [67]. Bitter tastes are usually disliked and avoided [65]. Nonetheless, this was not detected in the rest of the menu items. Thus, we can infer that the bitterness was due to arugula itself and not from sorghum. One of the main obstacles for whole grain acceptance is texture, along with flavor [68]. We confirmed this, since flavor was reported with higher frequency for the sorghum shrimp grits and spiced sorghum cookie, placing them as the first and second most accepted menu items.

The bivariate analysis revealed factors that influenced sorghum acceptance. Gender and college year demonstrated a significant association with the acceptance of the sorghum-based foods, indicating them as influential factors on the foods’ acceptance. Food selection and dietary patterns among college students are influenced by many factors [6]. Transitioning from being parent-dependent to being independent and being introduced to a food environment in which unhealthy food options prevail affect students’ food choices, making unhealthy diets predominant among college students [69]. Our results show that freshmen had lower (but not significant) acceptance of sorghum-based foods compared to other college years. This could be explained by the fact that freshmen are more prone to developing unhealthy eating habits resulting in fast food selection [70,71] due to their limited knowledge of healthy foods and their lack of cooking skills [70]. This finding was also reported by Yu and Tan’s study [72]. In their research, 50% of freshmen (n = 764) in a 4-year college reported consuming fast food or foods high in fat three or more times a week. Similarly, a previous study [73] confirmed that freshmen experienced changes in their nutrition, leading to a lower-quality diet and weight gain.

Even though food selection is dependent on sensory attributes such as appearance and texture, different factors also affect the acceptance of a new food item or food crop, with gender being one of them [74]. Our findings align with those of Lombardo, M. et al.’s study [75] where gender food preferences were significant, and females had a higher acceptance for whole grains and cereals among other foods compared to their male counterparts. In contrast, males had a higher intake of meat. These differences are attributed to physiological and sociocultural factors [75].

To ensure that the likeliness and acceptance were attributed to the sorghum-based foods themselves, gender and college year were adjusted in the General Linear Model to provide a more robust outcome about the foods’ acceptance. This was confirmed by the pairwise comparison shown in Table 5.

### 4.4. Eating and Purchase Intentions: Positive and Significant Relationships with Sorghum Consumption

Positive, significant relationships were found among overall acceptance, eating intentions, and purchase intentions across all of the sorghum-based foods. Nevertheless, the strength of these relationships varied across the foods. For instance, despite obtaining the lowest overall acceptance score, arugula salad with popped sorghum had a very strong relationship with eating intention (*r_s_* = 0.838, *p* < 0.01). On the other hand, the spiced sorghum cookie obtained the highest overall acceptance score, but it also had a strong relationship with eating intention (*r_s_* = 0.649, *p* < 0.01). These findings portray the complexity of consumer decision-making, where decisions are made beyond food acceptance such as social influence, exposure, and food stereotypes [76].

Purchase decisions are typically made based on the evaluation of product attributes, with some having a greater impact than others [77]. Most of the sorghum-based foods exhibited strong relationships between eating and purchase intentions, meaning that if people like to eat sorghum-based menu items, they tend to purchase them. Evidence from published studies suggests that the main factor that influences purchase intention among young adults is taste, agreeing with our results. Convenience is another major determinant for food purchasing for the same population [6,78]. Another key factor that was noted by Dhillon et al.’s study [55] for purchasing unhealthy foods over a healthy selection is price. Although we did not assess the relationships among convenience, price, and purchase intention, the selection of sorghum-based foods was based on the easiness of their replicability.

Our results point out thoughtful implications for sorghum-based foods in various ways including product development, public education, and market positioning. Although the current whole grain consumption does not meet the recommendation, the acceptance of sorghum-based foods was high enough to motivate sorghum purchase. Sensory elevation outcomes even conquer the hard texture of sorghum when sorghum-based foods are accommodated with different materials (such as meats) by enriching flavor and removing bitterness. These outcomes can be essential guidelines for new sorghum product development in any food-related places, such as home cooking, campus dining, restaurants, and food retail. With the uncertainty of public awareness of sorghum as a human food, our study outcomes draw attention to the need for consumer education about sorghum nutrition. This can be accomplished through school nutrition programs and/or governmental food assistant programs. Recently, the United States Department of Agriculture (USDA) added sorghum into the U.S. Food Buying Guide for Child Nutrition Programs so that school food service directors can create sorghum-based foods for school-aged children. Also, sorghum was included into the Women, Infant, and Children (WIC) food package. Due to this new policy, sorghum consumption that starts in school-aged children can lead to healthy eating outcomes like meeting DGA’s recommendations for whole grain consumption and reducing the risks of chronic diseases. Finally, our results can be used as a foundation for setting a market strategy for future sorghum-based foods by reflecting intentions to buy sorghum-based foods, gender preference, and differentiations with other gluten-free products.

## 5. Conclusions

This study investigated the acceptance of various sorghum-based foods and explained the relationships among overall acceptance, eating intention, and purchase intention among college students in a southern university in the U.S., and may be the first to report on these findings. The sorghum-based foods were accepted by the college students. It has been proven that knowing about the health benefits of a food product may increase eating and purchase intentions. Our study reflects the null consumption of sorghum. Therefore, through the development of innovative sorghum-based foods, exposure to and public education about sorghum as human food may significantly contribute to the increased consumption of sorghum whole grains. In addition, the acceptance of sorghum-based foods sets the foundation for future studies to assess sorghum’s potential health benefits for public health and wellbeing.

This study, however, has several limitations. All sorghum-based foods were cooked under different conditions with different ingredients. This could have affected the sensory attributes and perceptions of the food items. When developing sorghum-based recipes, we did not consider the use of food additives such as enzymes or combinations of other gluten-free grains to enhance sorghum’s texture. This will be addressed in future research. Since we focused on college students at a single (4-year) university, the generalization of this study’s outcomes to a broader population of university students may differ, suggesting representative sampling for future study. Understanding sorghum nutrition was not included in this study because this study focused on consumers’ acceptance of and intentions to purchase/eat sorghum as part of their diets based on the sensory evaluations of eight sorghum-based foods. In addition to sensory acceptance, public awareness and knowledge about sorghum nutrition can play a major role in promoting sorghum as a staple food, emphasizing the importance of consumer education about sorghum nutrition along with collaborations among stakeholders. Because sorghum is a sustainable and nutritious whole grain, its utilization as part of the food system and its impact on food security in low-income countries will be also studied in the future.

## Figures and Tables

**Table 1:** Eight selected sorghum foods, food type, and cooking method.

Food (Type)	Sorghum Format	Cooking Method
Sorghum Shrimp Grits (Main dish)	Ground	Ground sorghum was added to boiling water until it was cooked to the desired texture. Salted butter and cheese were added. Tomatoes with salted butter were sautéed to make the sauce. Lastly, shrimps were incorporated once cooked for 15 min or until the internal temperature reached 145 °F. Sorghum grits were topped with shrimps.
Arugula Salad with Popped Sorghum (Salad)	Intact popped grain	Sorghum grain was heated (medium heat) in a pan with olive oil. Once all grains were coated with oil, the pan was covered, and the sorghum was cooked. The pan was removed from the heat when the pops slowed to 1–2 pops every 5 s. Lastly, the grains were sprinkled with salt. Salad dressing was mixed with arugula leaves and topped with the popped sorghum.
Beef Sorghum (Main dish)	Intact grain	Meat was browned with oil. Vegetables were sautéed for 5 min, and tomato paste was incorporated along with sautéed garlic. Broth and spices were poured, while brought to a simmer; it was covered and cooked for approximately 55 min until the beef was tender. Lastly, the sorghum was added and cooked for another 45–60 min.
Sorghum Vegan Chili (Main dish)	Intact grain	In a large soup pot, oil was heated on a medium/high heat. Onion was cooked for 5 min, and sorghum was added and cooked for 3 min. Chili powder, tomato paste, and spices were stirred in, and drained beans and tomato sauce were incorporated and brought to a slow boil, simmered, and cooked for 15 min.
Sweet Sorghum Grits (Breakfast)	Ground	Ground sorghum was added to boiling water, and then slowly stirred. Once the sorghum had absorbed the water, it was seasoned.
Spiced Sorghum Cookie (Dessert)	Flour	Butter, sugar, sorghum syrup, and spices were mixed, and sorghum flour was gradually poured in until a homogeneous dough was obtained. The dough was rolled into balls and baked at 350 °F for 10 min until golden brown and slightly puffed.
Kale Sorghum Soup (Soup)	Intact grain	Sorghum was heated over a medium/high heat in a saucepan. Oil, onions, and garlic were added, seasoned, and cooked until softened. Kale was cooked until it wilted. Half of the white beans were smashed and added along with whole beans, whole sorghum, and the broth. After boiling, the saucepan was covered and the soup simmered for 20 min before being topped with grated cheese.
Chicken with Sorghum (Main dish)	Intact grain	After seasoning the chicken, an Instant pot was set to sauté, and oil and the chicken were added. After the meat was browned, it was removed and set aside. Seasoned vegetables were added and sautéed. Wine, chicken broth, and whole sorghum were added and cooked for 20 min.

**Table 2:** Demographic information and use of on-campus dining options among study participants.

Variable		N (%)
Gender	Female	48 (57.8)
	Male	35 (42.2)
Ethnic group	White, Non-Hispanic, or Latino	26 (31.3)
	Hispanic or Latino	22 (26.5)
	Asian	21 (25.3)
	Other	14 (16.9)
Age (years)	Under 20	43 (51.8)
	20 or over	40 (48.2)
College year	Freshman	32 (38.5)
	Sophomore	17 (20.5)
	Junior	15 (18.1)
	Senior	19 (22.9)
Dining service usage	Never	13 (15.7)
	Daily	36 (43.4)
	Weekly	18 (21.7)
	Monthly	11 (13.2)
	Not reported	5 (6.0)

N = 83.

**Table 3:** Sensory acceptance score of the eight sorghum-based menu items.

Sorghum-Based Food	Mean ± SD	Median
Chicken with sorghum	73.61 ± 14.46	74.60
Sorghum shrimp grits	74.51 ± 19.42	79.37
Arugula salad with popped sorghum	60.37 ± 16.77	61.90
Beef sorghum	73.36 ± 14.44	73.02
Sorghum vegan chili	68.16 ± 16.38	71.43
Sweet sorghum grits	65.54 ± 17.37	66.67
Spiced sorghum cookie	77.95 ± 14.23	80.95
Kale sorghum soup	65.25 ± 15.61	68.25

**Table 4:** Relationship between acceptance score and sorghum-based menu items after adjusting for the effect of gender and college year.

Parameter	β (95% CI)	t Value	*p*-Value
Menu item			
Kale sorghum soup (Reference)			
Chicken with sorghum	8.725 (4.033, 13.417)	3.651	<0.001 **
Sorghum shrimp grits	9.622 (4.930, 14.314)	4.027	<0.001 **
Arugula salad with popped sorghum	−4.881 (−9.588, −0.174)	−2.036	0.042 *
Beef sorghum	8.479 (3.787, 13.172)	3.548	<0.001 **
Sorghum vegan chili	2.844 (−1.863, 7.550)	1.186	0.236
Sweet sorghum grits	0.317 (−4.390, 5.023)	0.132	0.895
Spiced sorghum cookie	13.015 (8.323, 17.708)	5.447	<0.001 **
Gender			
Female (Reference)			
Male	−3.162 (−5.607, −0.717)	−2.54	0.011 *
College year			
Senior (Reference)			
Freshman	−2.917 (−6.016, 0.183)	−1.848	0.065
Sophomore	1.802 (−1.831, 5.436)	0.974	0.33
Junior	4.101 (0.391, 7.810)	2.171	0.03 *

* Significance at *p* < 0.05; ** significance at *p* < 0.01.

**Table 5:** Post hoc test on acceptance score according to sorghum-based foods for pairwise comparisons.

**Menu Item**	A	B	C	D	E	F	G	H
A	1							
B	1	1						
C	<0.001 **	<0.001 **	1					
D	1	1	<0.001 **	1				
E	0.39	0.132	0.037 *	0.522	1			
F	0.013 *	0.003 **	0.854	0.019 *	1	1		
G	1	1	<0.001 **	1	0.001 **	<0.001 **	1	
H	0.008 **	0.002 **	1	0.012 *	1	1	<0.001 **	1

Numbers in Table 5 represent *p*-values of post hoc test; * significance at *p* < 0.05; ** significance at *p* < 0.01; (A) chicken with sorghum, (B) sorghum shrimp grits, (C) arugula salad with popped sorghum, (D) beef sorghum, (E) sorghum vegan chili, (F) sweet sorghum grits, (G) spiced sorghum cookie, and (H) kale sorghum soup.

**Table 6:** Overall acceptance and eating and purchase intention correlations.

Menu Item	Overall Acceptance	Eating Intention	Purchase Intention
Chicken with sorghum			
Overall acceptance	1		
Eating intention	0.633 **	1	
Purchase intention	0.731 **	0.772 **	1
Sorghum shrimp grits			
Overall acceptance	1		
Eating intention	0.865 **	1	
Purchase intention	0.864 **	0.886 **	1
Arugula salad with popped sorghum			
Overall acceptance	1		
Eating intention	0.838 **	1	
Purchase intention	0.742 **	0.790 **	1
Beef sorghum			
Overall acceptance	1		
Eating intention	0.706 **	1	
Purchase intention	0.756 **	0.773 **	1
Sorghum vegan chili			
Overall acceptance	1		
Eating intention	0.771 **	1	
Purchase intention	0.758 **	0.791 **	1
Sweet sorghum grits			
Overall acceptance	1		
Eating intention	0.824 **	1	
Purchase intention	0.778 **	0.850 **	1
Spiced sorghum cookie			
Overall acceptance	1		
Eating intention	0.649 **	1	
Purchase intention	0.653 **	0.724 **	1
Kale sorghum soup			
Overall acceptance	1		
Eating intention	0.749 **	1	
Purchase intention	0.696 **	0.665 **	1

** Significance at *p* < 0.01.

## Data Availability

The original contributions presented in the study are included in the article, further inquiries can be directed to the corresponding author.
